# Osteogenic differentiation of amniotic epithelial cells: synergism of pulsed electromagnetic field and biochemical stimuli

**DOI:** 10.1186/1471-2474-15-271

**Published:** 2014-08-11

**Authors:** Qian Wang, Wenchao Wu, Xiaoyu Han, Ai Zheng, Song Lei, Jiang Wu, Huaiqing Chen, Chengqi He, Fengming Luo, Xiaojing Liu

**Affiliations:** Center of Rehabilitation Medicine, West China Hospital, Sichuan University, Chengdu, Sichuan P. R. China; Rehabilitation Key Laboratory of Sichuan Province, Chengdu, Sichuan P. R. China; Laboratory of Cardiovascular Diseases, Regenerative Medicine Research Center, West China Hospital, Sichuan University, Chengdu, Sichuan P. R. China; Sichuan University-The Hong Kong Polytechnic University Institute for Disaster Management and Reconstruction, Sichuan University, Chengdu, 610041 China; Department of Obstetrics and Gynecology, West China Second Hospital, Sichuan University, Chengdu, Sichuan P. R. China; Department of Pathology, West China Hospital, Sichuan University, Chengdu, Sichuan P. R. China; Laboratory of Biomedical Engineering, West China Center of Medical Sciences, Sichuan University, Chengdu, Sichuan P. R. China; Golden Card Ward, West China Hospital, Sichuan University, Chengdu, Sichuan P. R. China

**Keywords:** Amniotic epithelial cells, Osteogenic differentiation, Pulsed electromagnetic field, BMP-2, Wnt/β-catenin signaling, Reactive oxygen species, Integrinβ1

## Abstract

**Background:**

Pulsed electromagnetic field (PEMF) is a non-invasive physical therapy used in the treatment of fracture nonunion or delayed healing. PEMF can facilitate the osteogenic differentiation of bone marrow mesenchymal stem cells *in vitro*. Amniotic epithelial cells (AECs) have been proposed as a potential source of stem cells for cell therapy. However, whether PEMF could modulate the osteogenic differentiation of AECs is unknown. In the present study, the effects of PEMF on the osteogenic differentiation of AECs were investigated.

**Methods:**

AECs were isolated from amniotic membrane of human placenta by trypsin digestion and were induced by PEMF and/or osteo-induction medium. After 21 days we used real time RT-PCR and immunocytochemistry to study the expression of osteoblast markers. The signal transduction of osteogenesis was further investigated.

**Results:**

The PEMF stimulation, or osteo-induction medium alone could induce osteogenic differentiation of AECs, as shown by expression of osteoblast specific genes and proteins including alkaline phosphatase and osteocalcin. Furthermore, a combination of PEMF and osteo-induction medium had synergy effects on osteogenic differentiation. In our study, the gene expression of BMP-2, Runx2, β-catenin, Nrf2, Keap1 and integrinβ1 were up-regulated in the osteogenic differentiation of AECs induced by PEMF and/or osteo-induction medium.

**Conclusions:**

Combined application of PEMF and osteo-induction medium is synergistic for the osteogenic differentiation of AECs. It might be a novel approach in the bone regenerative medicine.

## Background

Amniotic epithelial cells (AECs), derived from the placenta, possess several advantages over both embryonic stem cells (ESCs) and adult stem cells. They express ESCs markers such as SSEA-1, SSEA-4 and Oct-4, and have the ability to differentiate into all three germ layers *in vitro*[1, 2]. Therefore, AECs have been proposed to be a good candidate for cell transplantation and regenerative medicine [3, 4].

AECs can be induced to differentiate into osteoblasts *in vitro* by treating with osteo-induction medium, which contains biochemical factors including dexamethasone, β-glycerol phosphate and ascorbic acid [5]. However, efficient induction of AECs differentiation into osteoblasts remains a challenge, and its mechanism is not fully understood.

Pulsed electromagnetic field (PEMF), a non-invasive physical treatment, is now used clinically to promote bone healing for fracture nonunion or delayed fracture healing [6, 7]. PEMF has various biological functions and can affect bone metabolism. Several studies have shown that PEMF can facilitate the osteogenesis by its direct effects on osteoblasts [8, 9]. Recently, it has been reported that PEMF with specific parameters could modulate osteogenic differentiation of bone marrow derived mesenchymal stem cells (BMMSCs) *in vitro*[10–12]. These findings suggest that PEMF might be able to induce AECs to differentiate into osteoblasts.

In this study, we tested the hypothesis that PEMF could modulate the osteogenic differentiation of AECs. We hypothesized that physical force (PEMF) and biochemical treatment and/or their combination might play important roles in the process of osteogenic differentiation of AECs. We explored the possible mechanisms, especially the regulating role of BMP-2 and ROS pathways in the process.

## Methods

### AECs isolation, culture and identification

Five placenta samples through cesarean delivery were collected aseptically with the informed consents of parturients. Primary cultures of human AECs were isolated from amniotic membrane by trypsin digestion method and cultured in standard culture medium, according to Miki’s methods [1]. AECs were identified by the specific epithelial cell marker cytokerin 19 using immunocytochemistry. Phenotype of human AECs was analyzed by flow cytometry.

The study was conducted according to the Declaration of Helsinki and approved by the medical ethics committee of the West China Hospital, Sichuan University.

### PEMF stimulation

PEMF was generated by a commercial, clinically approved PEMF system (Model XT-2000B). During each pulse, the applied field increased from 0 to 1mT in 1.5 ms and then decayed back to 0 in 5 ms. The 50Hz repetitive pulsed waveform was based on other previous investigations [13, 14]. AECs from the third to fifth passages were maintained in standard culture medium as controls or in osteo-induction medium (OM) [5]. For PEMF treatment, the cells were exposed daily to 50Hz 1mT PEMF stimulation for 30 minutes each time and twice a day, with an interval of 12 hours. The treatment lasted for 21 days. Each experiment was in three replicates.

### Detection of alkaline phosphatase (ALP) activity by histochemistry staining

The activity of ALP was detected by histochemistry staining using a BCIP/NBT ALP kit (R&D Biotech, UK) according to manufacturer’s instructions [15]. The percentage of ALP-positive cells was calculated by ImageJ software (National Institutes of Health, USA).

### Detection of osteocalcin (OC) protein expression by immunocytochemistry

AECs were plated onto coverslips in 6-well plates and treated with different stimuli. The protein expression of OC was measured by immunocytochemistry as described before [16] Images were captured on a Leica DFC 300FX Digital Camera system (Leica, UK) and analyzed with the ImageJ software to semi-quantitatively determine the level of cytoplasmic OC.

### Assessment of calcium deposition by alizarin red staining

Calcium deposition, one of the markers of osteogenic differentiation of stem cell, was assessed by alizarin red S staining [17]. Images were captured on a Leica DFC 300FX Digital Camera system (Leica, UK). The amount of calcified deposition was semi-quantitatively calculated with ImageJ software.

### Quantitative reverse transcription PCR (Real-time RT-PCR)

Total RNA was extracted from AECs with TRIZOL (Invitrogen, USA), and cDNA was synthesized using a reverse transcription (RT) kit (Toyobo, Osaka, Japan) [16]. Quantitative PCR was carried out on BIO-RAD CFX96™ Real-Time PCR Detection System with fluorescence dye EvaGreen (EvaGreen Supermix kit, Bio-Rad, USA). Primer sequences are shown in Table 1. Data analysis was carried out by Bio-Rad software using relative quantification. The comparative cycle-threshold method (2^-△△CT^) was used for quantification of gene expression [17]. For quantification, the target sequence was normalized to the β-actin mRNA levels.

**Table 1:** **Primer sequences and amplicon sizes for real-time RT-PCR**

Genes	Gene Bank ID	Primer Sequence (5'-3')	Amplicon (bp)
**ALP**	NM_000478	F: 5' GGAACTCCTGACCCTTGACC 3'	86
		R: 5' TCCTGTTCAGCTCGTACTGC 3'	
**OC**	NM_001199662	F:5' AGGGCAGCGAGGTAGTGAA 3'	151
		R: 5' TCCTGAAAGCCGATGTGGT 3'	
**BMP-2**	NM_001200	F:5' TCAAGCCAAACACAAACAGC 3'	103
		R: 5' AGCCACAATCCAGTCATTCC 3'	
**RUNX2**	NM_001015051	F:5' TTACTTACACCCCGCCAGTC 3'	139
		R: 5' TATGGAGTGCTGCTGGTCTG 3'	
**β-catenin**	NM_020248	F:5' CCTATGCAGGGGTGGTCAAC 3'	95
		R: 5' CGACCTGGAAAACGCCATCA 3'	
**Integrinβ1**	NM_002211	F:5' CAAGCAGGGCCAAATTGTGG 3'	121
		R: 5' TGTCATCTGGAGGGCAACCC 3'	
**Nrf2**	NM_001145413	F:5' ATTGCCTGTAAGTCCTGGTCA 3'	182
		R: 5' ACTGCTCTTTGGACATCATTTCG 3'	
**Keap1**	NM_203500	F:5' GTGGCTGTCCTCAATCGTCT 3'	127
		R: 5' GGATGGTGTTCATTGCTGTG 3'	
**β-actin**	NM_001099771	F:5' ACTATCGGCAATGAGCGGTTC 3'	77
		R: 5' ATGCCACAGGATTCCATACCC 3'	

### Statistical analysis

The experimental data were presented as means ± SD. Group means were compared by *One-way ANOVA* using the statistical software SPSS 10.0 for Windows (Chicago, IL, USA), and *P* value < 0.05 was considered to be statistically significant.

## Results

### Characterization and phenotype of isolated AECs

**Figure 1:**
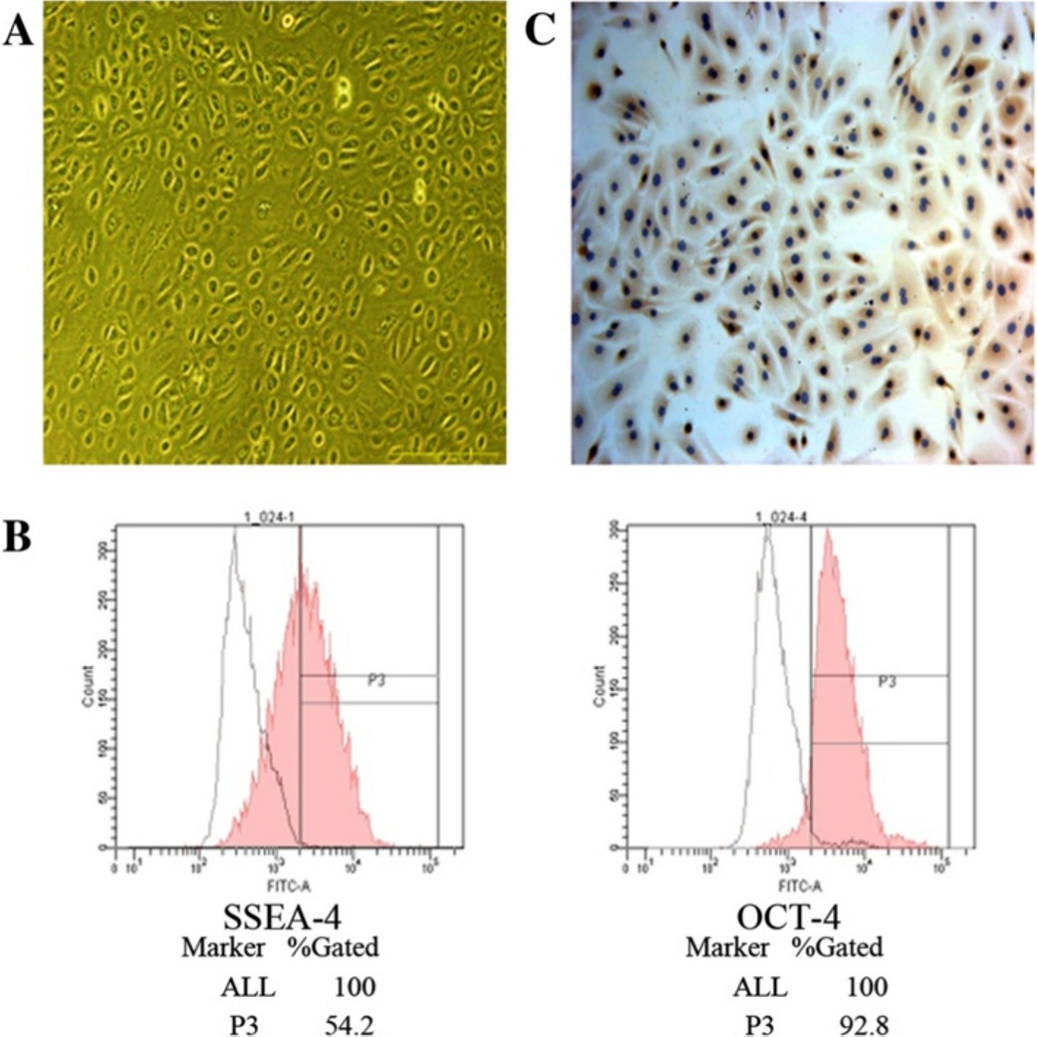
**Characterization and phenotype of isolated Amniotic epithelial cells (AECs). (A)** Primary culture of human AECs. AECs were successfully isolated from human term placenta and formed a confluent monolayer of cobblestone-shaped epithelial cells after 3 days of culture in the standard culture media (magnification of 200×). **(B)** Surface antigen SSEA-4 and the pluripotency marker Oct-4 in primary cultured AECs. Flow cytometry revealed the presence of SSEA-4 (54.2%) and Oct-4 (92.8%) in primary cultured AECs. The open peaks show the isotype-matched antibody control. **(C)** Specific marker of epithelial cells identified by immunocytochemistry. Primary cultured AECs could express cytokeratin 19, the epithelial cell marker (magnification of 200×).

Amniotic epithelial cells (AECs) were successfully isolated from human placenta and formed a confluent monolayer of cobblestone-shaped epithelial cells after 3 days of culturing in the standard culture media (Figure 1A).

In our study, flow cytometry analysis revealed that 54.2% of primary cultured AECs were positive for SSEA-4 and 92.8% were positive for Oct-4 (Figure 1B). Furthermore, the AECs could also express cytokeratin 19, the epithelial cell marker (Figure 1C). Consistent with previous reports [2, 5], we provided further evidence that AECs expressed not only the epithelial cell marker cytokeratin 19, but also the stem cell markers such as SSEA-4 and Oct-4.

### PEMF modulates the osteogenic differentiation of AECs

In order to examine whether PEMF could play a role in osteogenic differentiation of AECs, we used real time RT-PCR and immunocytochemistry to study the expression of osteoblast markers, such as alkaline phosphatase (ALP) and osteocalcin (OC) in AECs followed by (1) standard culture media (control group), (2) administration of PEMF for AECs cultured in standard culture media (PEMF only), (3) osteo-induction medium (OM only), (4) combined treatments with PEMF and osteo-induction medium for various time points (up to 21 days).

**Figure 2:**
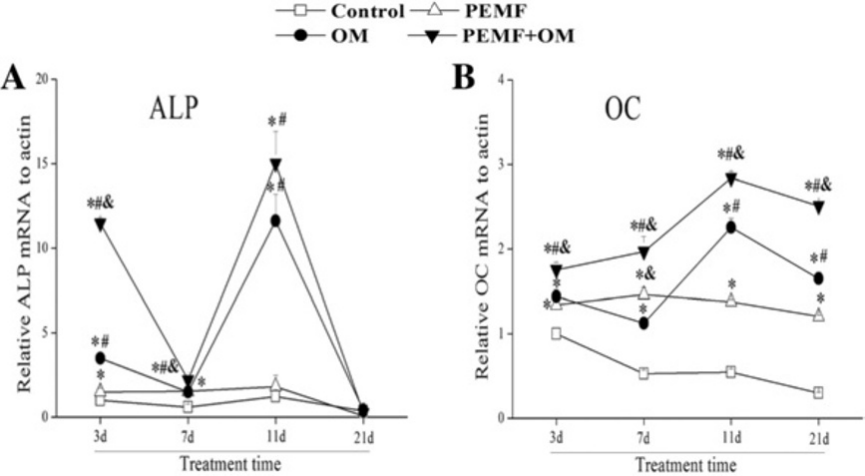
**Combined treatment of PEMF and osteo-induction medium increased ALP (A) and OC (B) mRNA expression.** Experiments were performed three times with the similar results (n = 3 in each group). * indicates *P* < 0.05 *vs* Control, ^**#**^ indicates *P* < 0.05 *vs* PEMF and & indicates *P* < 0.05 *vs* OM. Abbreviations: Control, standard culture media; PEMF, PEMF exposure; OM, osteo-induction medium; PEMF + OM, combined treatments with PEMF and osteo-induction medium; ALP, alkaline phosphatase; OC, osteocalcin.

Real time RT-PCR results showed that application of PEMF alone increased the gene expression of ALP and OC above basal levels after 7 days of treatment. The osteo-induction medium alone induced the gene expression of ALP and OC at day 3 post-treatment. The combined induction of ALP and OC mRNA expression were both peaked at day 11, and the elevated level lasted up to day 21 after the combined treatments (Figure 2).

**Figure 3:**
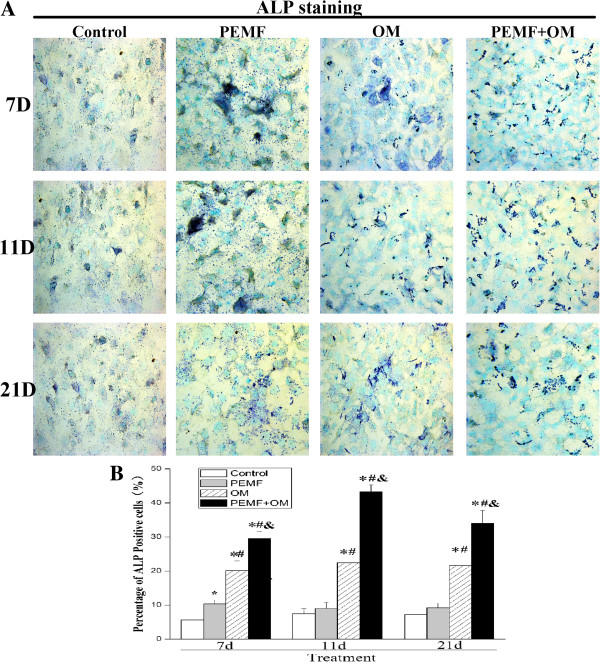
**ALP activity was increased during the osteogenic differentiation of AECs. (A)** The ALP activity was detected by BCIP/NBT staining (magnification of 400×). **(B)** The percent of ALP-positive cells (containing blue, insoluble, granular dye deposit) was calculated by ImageJ software Experiments were performed three times with the similar results (n = 3 in each group). * indicates *P* < 0.05 *vs* Control, ^**#**^ indicates *P* < 0.05 *vs* PEMF and & indicates *P* < 0.05 *vs* OM.

**Figure 4:**
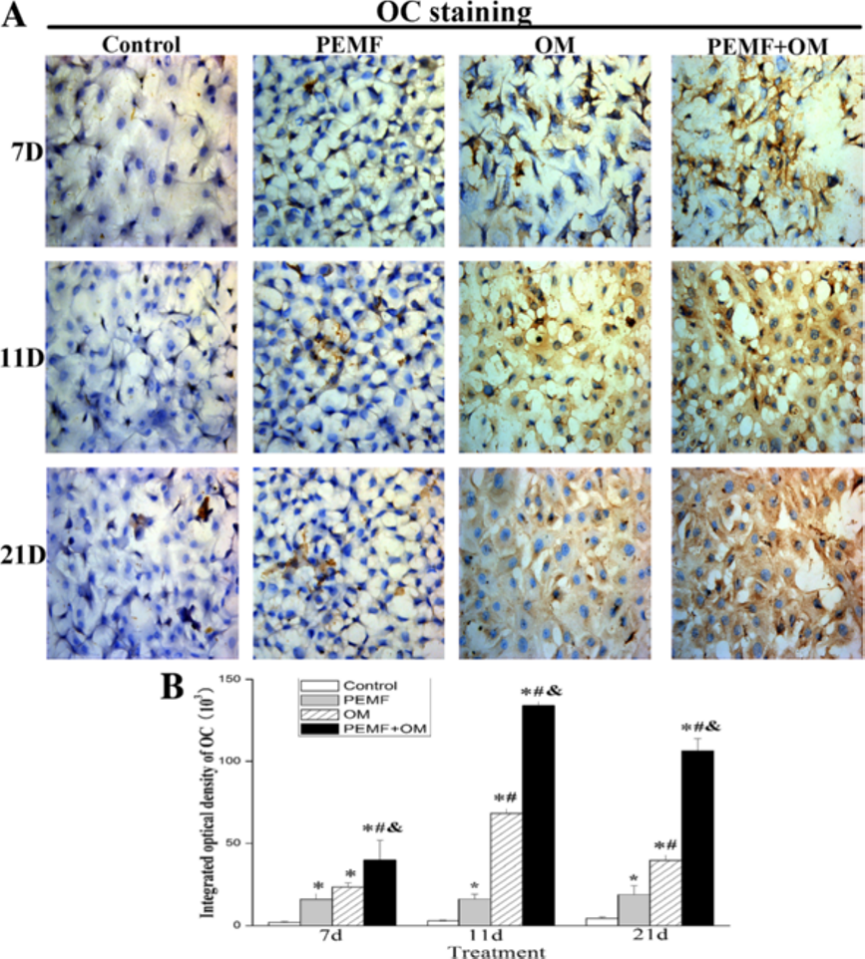
**OC protein expression was induced to rise during the osteogenic differentiation of AECs. (A)** The expression of OC protein was analyzed by immunocytochemistry (magnification of 400×). **(B)** The integrated optical density (IOD) of OC was measured by ImageJ software. Experiments were performed three times with the similar results (n = 3 in each group). * indicates *P* < 0.05 *vs* Control, ^**#**^ indicates *P* < 0.05 *vs* PEMF and & indicates *P* < 0.05 *vs* OM.

Next, we examined the administration of PEMF and/or OM-induced change of ALP and OC protein level in AECs. Exposure of AECs to PEMF alone induced the ALP activity at day 7. Culture of AECs in osteo-induction medium increased the ALP activity, which reached the highest level at day 11, and the increased level lasted up to day 21 post-treatment. Moreover, combined application of PEMF and osteo-induction medium resulted in a much higher level of ALP activity compared with other groups (Figure 3). The expression of OC protein was analyzed by immunocytochemistry, which showed that AECs after combined stimulation of PEMF and osteo-induction medium presented a much stronger OC protein expression, than what either treatment alone (Figure 4).

**Figure 5:**
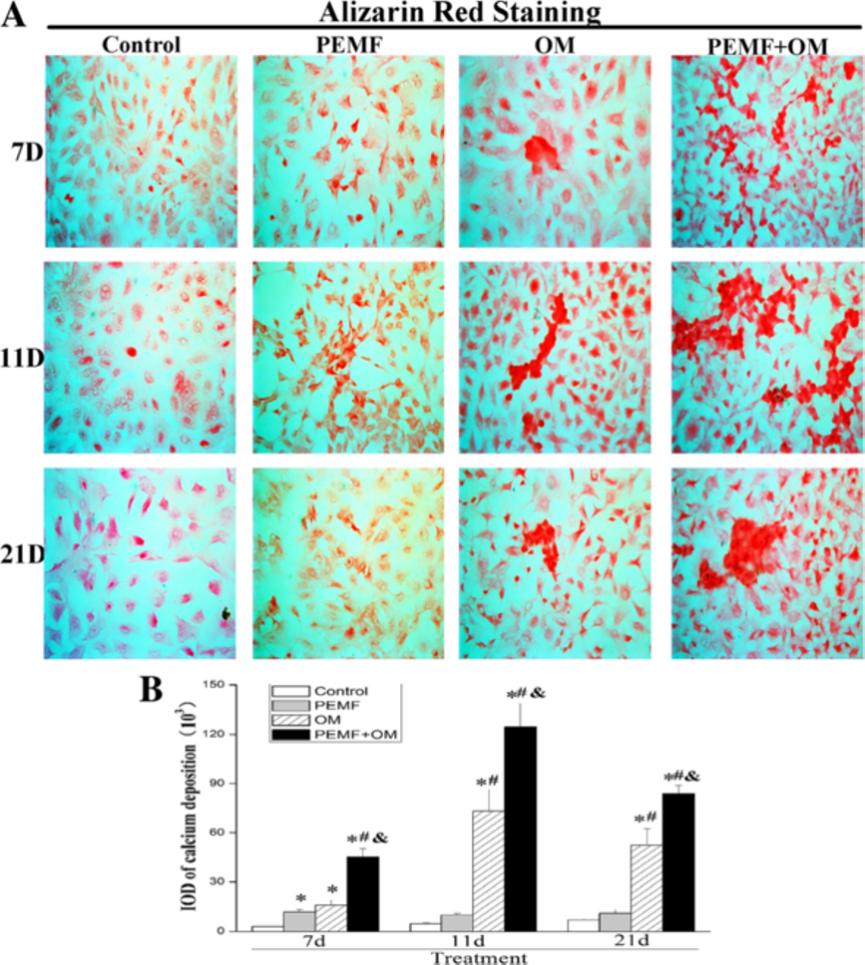
**A marked increase of calcium deposits was evoked during the osteogenic differentiation of AECs. (A)** Calcium deposition was assessed by alizarin red S staining (arrows) (magnification of 400×). **(B)** The amount of calcified deposition was semi-quantitatively calculated with ImageJ software. Experiments were performed three times with the similar results (n = 3 in each group). * indicates *P* < 0.05 *vs* Control, ^**#**^ indicates *P* < 0.05 *vs* PEMF and & indicates *P* < 0.05 *vs* OM.

The osteogenic differentiation of AECs was also validated by detection of the calcium deposits [17]. Alizarin red S staining results showed that, application of PEMF alone increased calcium deposits in AECs as early as day 7 after treatment. The osteo-induction medium also evoked a marked increase of extracellular matrix calcification in AECs. Furthermore, a combination of PEMF with osteo-induction medium induced much more calcium deposition in AECs than the other treatments, and the effect was peaked at day 11 (Figure 5).

Taken together, we demonstrated that a combination of PEMF and osteo-induction medium had much stronger effects on osteogenic differentiation of AECs, than what either treatment alone had.

### PEMF might modulate the osteogenic differentiation of AECs via BMP-2/Runx2 or Wnt/β-catenin signaling

**Figure 6:**
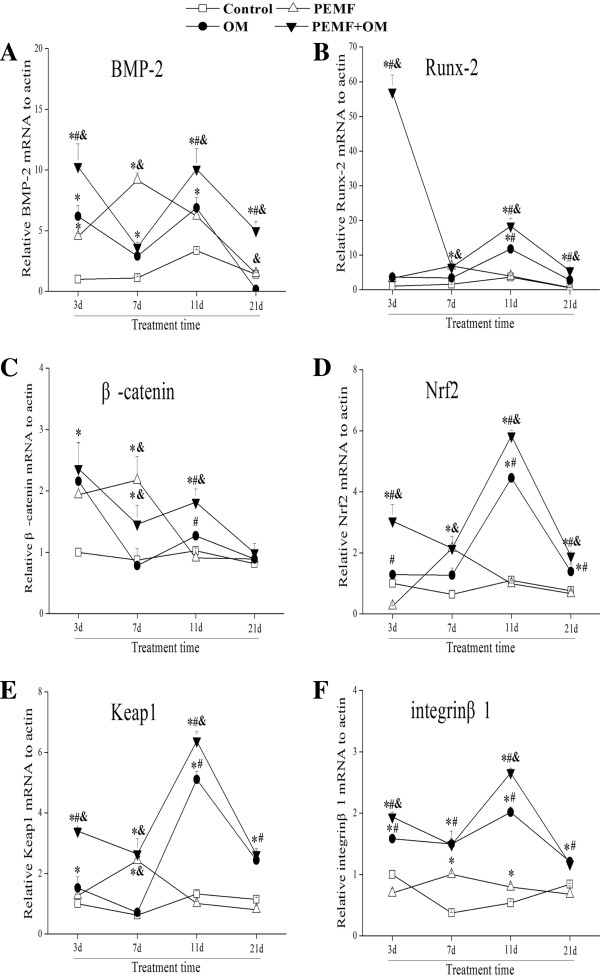
**Molecular mechanisms linked to PEMF-induced osteogenic differentiation of AECs.** Targeted genes involve: **(A)** bone morphogenetic protein 2 (BMP-2); **(B)** Runx-2; **(C)** β-catenin; **(D)** Nrf2; **(E)** Keap1; **(F)** Integrinβ1. Experiments were performed three times with the similar results (n = 3 in each group). * indicates *P* < 0.05 *vs* Control, ^**#**^ indicates *P* < 0.05 *vs* PEMF and & indicates *P* < 0.05 *vs* OM.

To investigate the possible role of BMP-2/Runx2 or Wnt/β-catenin signaling in osteoblast differentiation of AECs, we assessed the expression change of BMP-2, Runx2 and β-catenin mRNA in AECs with different treatments. Exposure of PEMF alone induced the expression of BMP-2, Runx2 and β-catenin above basal levels after 7 days of treatment. Osteo-induction medium alone promoted the expression of BMP-2, Runx2 and β-catenin, followed by a gradual decrease after treatment. Moreover, combined application of PEMF with osteo-induction medium led to a significant up-regulation in the expression of BMP-2, Runx2 and β-catenin compared with other treatments (Figure 6A-C).

### Nrf2 and Keap1 might be involved in the PEMF-induced osteogenic differentiation of AECs

Reactive oxygen species (ROS) could play vital role in the self-renewal and differentiation of stem cells [18, 19]. To test whether Nrf2 and Keap1, the master regulators of ROS generation, might be implicated in the osteogenic differentiation of AECs, we examined the gene expression of Nrf2 and Keap1. PEMF treatment alone significantly induced the expression of Nrf2 at day 7. Combined use of PEMF and osteo-induction medium evoked a significant up-regulation of Nrf2 mRNA expression compared with other treatments (Figure 2F). Furthermore, the gene expression profile of Keap1 was similar to that of Nrf2 during the osteogenic induction of AECs (Figure 6D,E).

### Integrinβ1 may be the PEMF sensitive receptor in the process of osteogenic differentiation of AECs

Integrinβ1 is a well-known mechanoreceptor in mediating the transduction of mechanical strain in most cells [20]. We therefore investigated whether integrinβ1 might also act as the PEMF-sensitive receptor in the osteogenic induction of AECs. Real-time RT-PCR results showed that, PEMF alone induced the significant up-regulation of gene expression of integrinβ1 in AECs at day 7. Moreover, treatments with PEMF and osteo-induction medium together evoked a significant up-regulation of integrinβ1 mRNA expression in comparison to the other treatments (Figure 6F). These results suggest that, integrinβ1 appears to be one of the PEMF or biochemical sensitive receptor mediated in the osteogenic differentiation of AECs.

## Discussion

In the present study, the combined effects of physical treatment (PEMF) and biochemical stimuli (osteo-induction medium) on the osteogenic differentiation of AECs were investigated. The major findings of this study are: (1) Combined application of PEMF and osteo-induction medium to AECs has stronger effects on osteogenic differentiation, than either treatment alone; (2) Activation of signaling pathways, such as BMP-2 and Wnt/β-catenin, might be important in mediating the PEMF and/or osteo-induction medium-induced osteogenic differentiation of AECs; (3) Nrf2/Keap1, master regulators of ROS, might be implicated in the PEMF and/or osteo-induction medium-induced osteogenic differentiation of AECs; (4) The expression of integrinβ1 mRNA was up-regulated in the process of osteogenic differentiation of AECs induced by PEMF and/or osteo-induction medium. Our data indicate that PEMF could play an important role in the modulation of osteogenic differentiation of AECs. These observations also establish possible links of integrinβ1 and Nrf2/Keap1 in mediating PEMF and/or osteo-induction medium-induced osteogenic differentiation of AECs.

PEMF could interfere with cellular growth, proliferation and differentiation, as recently demonstrated in osteoblasts [9]. PEMF is also capable of regulating Ca^2+^ homeostasis and promoting fracture healing [21]. There have been several studies to demonstrate the inductive effect of PEMF in the osteoblast differentiation of MSCs [11, 12, 14, 22, 23]. Tsai et al. reported that PEMF could induce early onset of osteogenic differentiation of MSCs on the basis of ALP activity and stimulate the gene expression of ALP and Runx-2 at day 7 but lower at day 10 in the process of osteogenic induction [22]. The similar results were reported by Sun et al. who found that exposure to PEMF significantly increased ALP gene expression during the early stages of osteogenesis and enhanced mineralization near the midpoint of osteogenesis [12]. Additionally, Song et al. revealed that PEMF could up-regulate the gene expression of Runx-2, bone sialoprotein (BSP) and osteopontin (OPN); enhance the alkaline phosphatase activity and calcium deposition in a time-dependent manner. Furthermore, MEK/ERK signaling pathway might be mediated in this process of osteogenic differences of MSCs [11].

The present study showed that PEMF stimulation alone could induce the expression of osteoblast markers ALP and OC at both gene and protein levels at specific time point(day 7). The osteo-induction medium was also able to induce the osteogenesis of AECs as reported in the previous literature [5]. Moreover, combined application of PEMF and osteo-induction medium led to significant up-regulation of ALP and OC expression and promoted the obvious extracellular matrix calcification. Our findings demonstrated the synergistic effects of physical (PEMF) and biochemical stimuli (OM) on the osteogenic induction of AECs. The effect of PEMF on the osteoblast differentiation of AECs may depend on the specific parameters of PEMF, such as waveform, duration, frequency and magnetic flux, as well as different cell types [22, 24]. This may be the reason why the effects of PEMF alone on the stimulation of osteogenic differentiation of AECs were found most obvious at day 7. Therefore, further studies are necessary to determine the optical parameters of PEMF in the osteogenic differentiation of AECs.

Among the intracellular signals involved in PEMF actions, the activation of bone morphogenetic proteins (BMPs) plays important roles. Runx2, as a downstream regulator of BMP-2 signaling, is necessary for osteoblast differentiation [25]. Wnt/β-catenin signaling is also of crucial importance for MSCs osteogensis [26]. Our results showed that the gene expression of BMP-2, Runx2 and β-catenin were all up-regulated in the osteogenic differentiation of AECs induced by PEMF alone at the specific time point (day 7). Osteo-induction medium alone or combined with PEMF exposure could induce BMP-2, Runx2 and β-catenin gene expression especially at the early stage of osteogenic induction (day 3). Additionally, similar gene expression profiles of BMP2, Runx2 and β-catenin were observed. These results may be due to the fact that both BMP-2/Runx2 and Wnt/β-catenin signaling could play important role in activating the osteogenic induction of AECs at the early-stage, while down-regulation of these signals are required for the late-stage of osteogenesis and matrix mineralization [26, 27]. Therefore, the present observations showed that PEMF and/or osteo-induction medium-induced the osteogenic differentiation of AECs may be via activation of both BMP-2 and Wnt/β-catenin signaling.

Another pathway implicated in the PEMF action is the generation of reactive oxygen species (ROS) [28, 29]. Recent study showed that Nrf2/Keap1, master regulator of ROS generation, would be required for intestinal stem cells (ISCs) proliferation [19]. In our study, the induction expression of Nrf2 and Keap1 were observed in the treatment of PEMF and/or osteo-induction medium, and the gene expression of Nrf2 and Keap1 exhibited similar profiles during the osteogenesis of AECs. As key regulators of ROS generation, Nrf2/Keap1 might be of potential importance during osteogenic differentiation of AECs induced by PEMF and/or the osteo-induction medium.

The mechanism involving how the cells sense and transduce physical stimulation such as PEMF into biochemical signals has remained elusive. Integrins function as one of the mechanoreceptors, which are capable of switching mechanical strain to biochemical signals [20, 30]. This process is comprised of binding to the extracellular matrix (ECM) ligands and activating the specific signaling pathways which would be involved in the mechanical-induced differentiation of cells [31]. Kasten et al. reported that certain biological functions of MSCs would be performed under the circumstance of integrin-mediated mechanical forces [32]. Franceschi et al. found that the application of mechanical force to osteoblasts could activate the MAPK signaling through integrin α2 and β1 [33]. However, little is known about the role of integrins in the differentiation of AECs. The current study showed that, integrinβ1 gene expression was up-regulated in the PEMF and/or osteo-induction medium-induced osteogenic differentiation of AECs. We propose that integrinβ1 might act as a receptor, which can be inducible in response to the physical stimulation, especially to the PEMF.

Our preliminary study demonstrates the role of BMP-2, Wnt/β-catenin, Nrf2/Keap1 and integrinβ1 in the osteogenic differentiation of AECs, only from the perspective of gene expression. Even though these molecules/pathways may be critical in mediating PEMF/osteo-induction medium enabled osteogenic differentiation, it is difficult to reveal the exact mechanisms without further testing, such as pathway-specific approaches. Therefore, additional experiments are needed to explore the mechanisms on how these signalings, ROS and integrinβ1 play role in regulating the PEMF-induced osteogenic differentiation of AECs.

## Conclusions

The present study demonstrates that combined use of physical (PEMF) and biochemical stimuli (osteo-induction medium) is synergistic for the osteogenic differentiation of AECs, which might be a novel approach in bone regenerative medicine. BMP-2/Runx2, Wnt/β-catenin, ROS and integrin signalings might be involved in the osteogenic differentiation of AECs induced by PEMF and/or osteo-induction medium.
